# Utilizing 3-Dimensional Cardiac Models With Point-of-Care Ultrasound Video Tutorials to Improve Medical Student Education: A Double-Blinded Randomized Control Study

**DOI:** 10.7759/cureus.34978

**Published:** 2023-02-14

**Authors:** Hillary McKinley, Heather Stuart, Shaina Ailawadi, Jordan Brunswick

**Affiliations:** 1 Emergency Medicine, Wright State University, Fairborn, USA; 2 Internal Medicine, Wright State University Boonshoft School of Medicine, Dayton, USA

**Keywords:** pocus, medical student, education, 3-dimensional, cardiac, ultrasound

## Abstract

Introduction

Ultrasound is a rapidly expanding imaging modality that many medical schools are incorporating into a structured curriculum. Learning both anatomy and ultrasound imaging simultaneously is intuitively challenging. This double-blinded, randomized control study examined the effect of utilizing three-dimensional (3d) cardiac models within an ultrasound video tutorial in order to achieve improved cardiac ultrasound anatomy education.

Methods

Thirty-nine (39) first- and second-year medical students at a single medical school voluntarily participated. The control group watched a video tutorial on cardiac ultrasound anatomy while the experimental group watched a similar video tutorial that also included a 3d cardiac model. The effect was measured with a multiple-choice test that included a sub-analysis of ultrasound principles. The test was unique in that no text or context clues were provided on the reference images, further challenging anatomic identification.

Results

The findings of the study included a p-value of 0.73 for the ultrasound principles section and a p-value of 0.77 for the cardiac anatomy. There was no statistical difference in the primary outcome or in the subgroup analysis. Post-hoc analysis demonstrated the study was underpowered.

Conclusions

This study is the first of its kind to utilize an innovative testing method that holds promise for future research in regards to utilizing 3d models with ultrasound education. The study was underpowered, therefore no definitive conclusions about the utility of 3d cardiac models in the educational process can be ascertained.

## Introduction

First and second-year medical students generally have a curriculum that focuses on gross anatomy and physiology. Cadaveric anatomy labs allow for the gross visualization of a static structure in relation to its surroundings. In more recent years, many medical schools have also incorporated ultrasound into their standardized curriculum as a way to enhance anatomy education [1]. In particular, the emergency medicine point-of-care cardiac ultrasound examination allows for the visualization of cardiac anatomy and physiology that would otherwise be unachievable in the cadaveric lab or with three-dimensional (3d) modeling alone. A PubMed literature search to assess articles and case reports utilizing both ultrasound and 3d modeling for educational purposes was conducted. There are no known prior studies of this nature.

The authors hypothesized that the group who viewed the video tutorial with the inclusion of a 3d cardiac model would score higher on the assessment than the group that did not receive instruction with a 3d model. A subgroup analysis within the test to assess the study subject's knowledge of ultrasound terminology and non-anatomic questions served as the internal validation. To this extent, it was hypothesized that there would be no statistical significance in the test scores between the two study arms in regard to the subgroup analysis of the ultrasound terminology and non-anatomic questions.

## Materials and methods

A double-blinded randomized control trial was developed. The Wright State University (WSU) Institutional Review Board (IRB) procedures and policies were followed in accordance with Protocol #07188 as an exempt research study. Prior to scheduling the data collection event, a calculation was performed to determine how many subjects would be needed to obtain appropriate statistical power, calculated as a population of 16 participants in each study arm [2]. The sample size was determined with a broad assumption. The authors assumed that the 3d group would score roughly 60% correct on their test, with a standard deviation of 3. This was intentionally large, as there is no known pilot study or other published literature describing similar testing. The best fit to help in this assumption describes a 5-point testing system based on the ability to acquire sonographic cardiac views to assist with anatomic identification [3].

**Figure 1:**
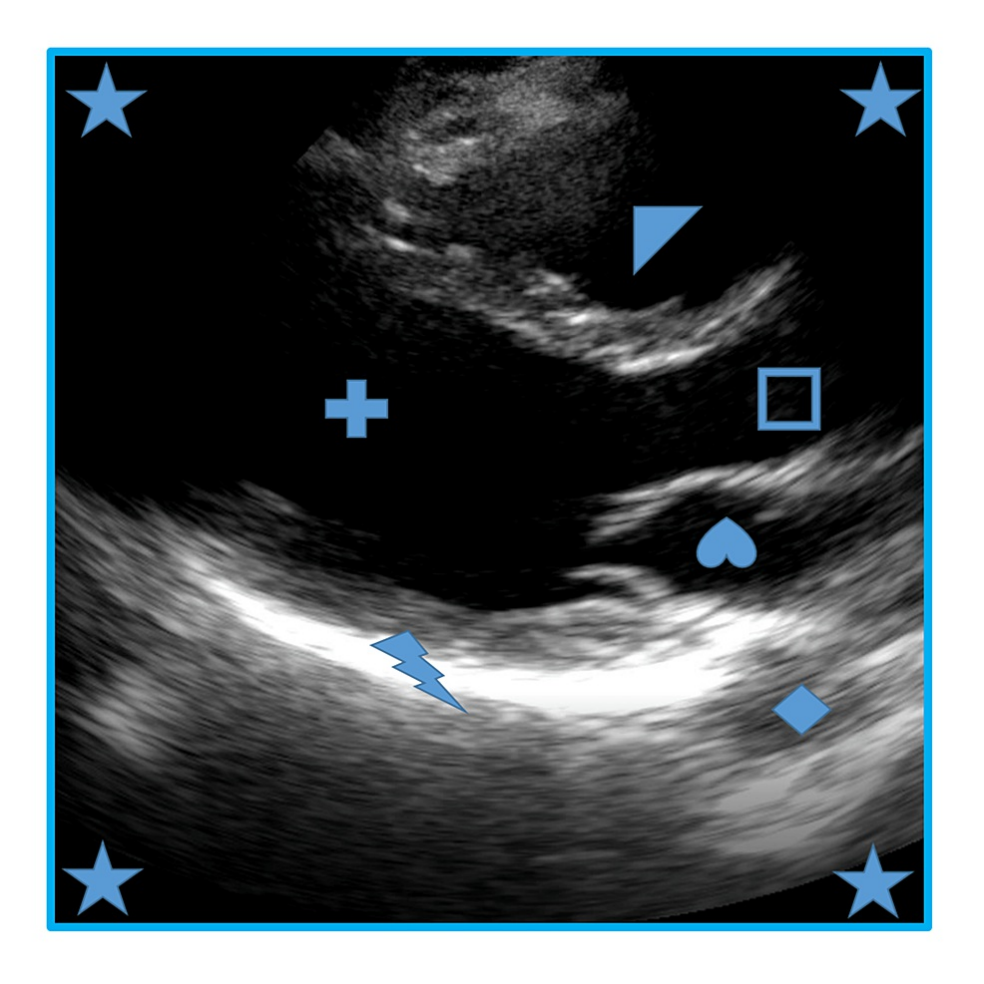
Example of the test reference image

**Figure 2:**
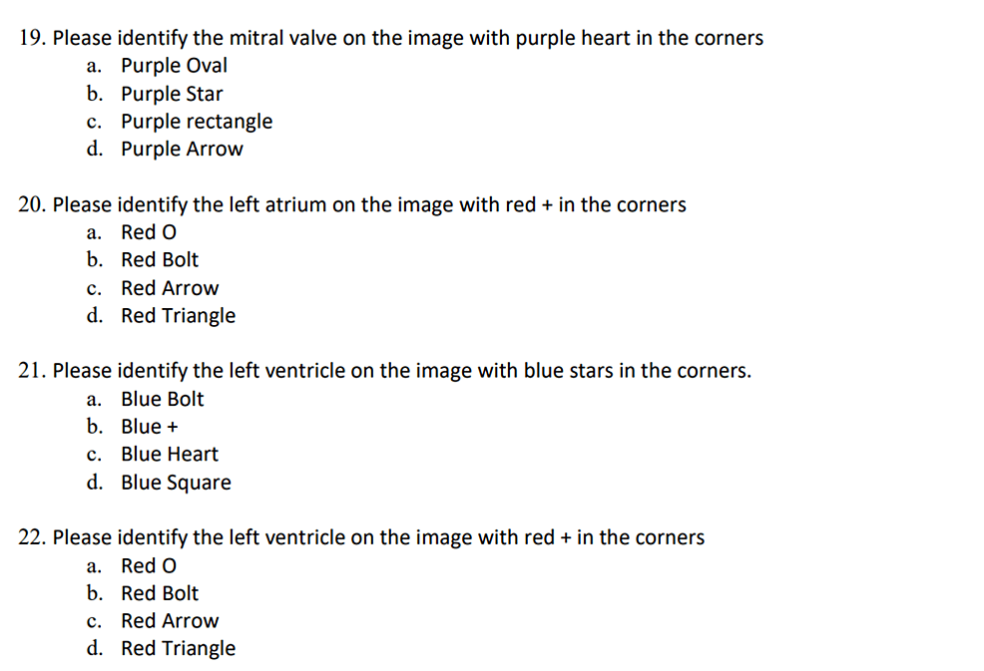
Example of cardiac anatomy test questions

The study participants were medical student volunteers. The total first and second-year medical school population of 257 students was sent an information sheet via email. Inclusion criteria required enrollment in the first or second year of medical school and having an electronic device capable of accessing the internet. Exclusion criteria included anyone with prior ultrasound experience other than curriculum requirements or anyone feeling unwell on the day of data collection due to health safety protocols. Students were provided pizza and non-alcoholic beverages in exchange for their voluntary participation. A total of 39 subjects agreed to voluntarily participate in this study. It is unknown how many of the first or second-year students were ineligible, as only those who voluntarily participated on the day of the study were screened for inclusion and exclusion criteria. The participation rate was 15.2%. No personal information about participants was collected. After screening for inclusion and exclusion criteria, the subjects were randomized via coin flip. One of the authors (SA) was unblinded and sent each participant the corresponding video file. All tests were completed, returned, graded, and analyzed by the remaining blinded authors using the group names “Heads” or “Tails.” Unblinding only occurred at the conclusion of the data analysis. The data were collected over two days. No study participants were duplicated. The 3d model used in the video tutorial was a Learning Resources® Cross-Section Heart Model. Test materials were printed and distributed to each participant. The images were printed on square paper in black and white, with color symbols used to label anatomic structures. No textual labels or orientation of the images were provided in an attempt to limit context clues (Figures 1-2, Videos 1-2).

**Video 1:** Control cardiac model ultrasound tutorial

**Video 2:** 3d cardiac model ultrasound tutorial

Of the 24-question, one-best answer, multiple-choice test, Questions #1-8 tested ultrasound principles while Questions #9-24 tested first-order cardiac anatomy. This test was created by the authors and did not undergo prior validation. Images were obtained by Ultrasound Fellowship Trained Faculty, with adequate views of the parasternal long axis, parasternal short axis, apical four-chamber, and subxiphoid images of the heart [4,5].

## Results

**Figure 3:**
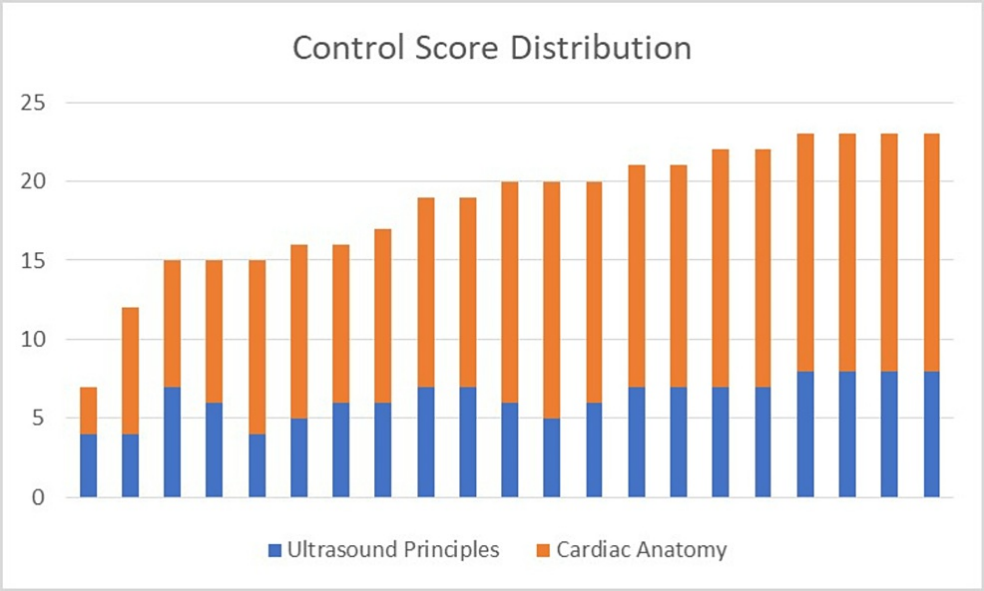
Control group score distribution

**Figure 4:**
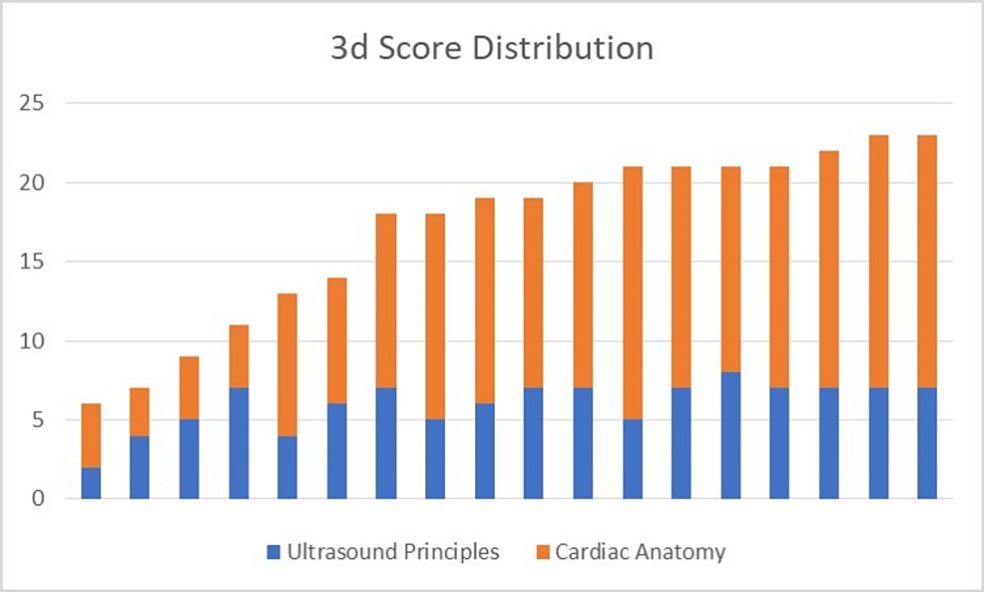
3d group score distribution

**Figure 5:**
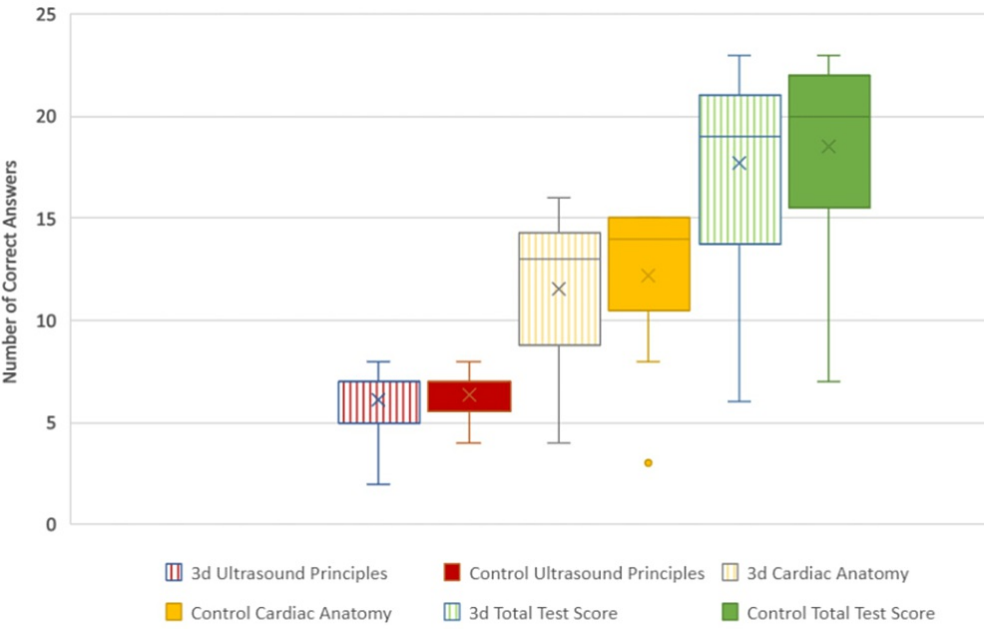
Box and whisker plot of control and 3d test scores

There was a total of 39 study participants, with 18 participants being assigned to the 3d group and 21 being assigned to the control group. The test scores for the two groups are graphically displayed in Figures 3, 4. Since the test scores were not normally distributed, a two-tailed Whitney Wilcoxon test was employed in this non-parametric setting. The statistical analysis program SAS v9.4 (SAS Institute Inc., Cary) was utilized by a WSU statistical consultant. The total and subgroup test scores are graphically displayed in Table 1. This is graphically displayed in Figure 5 as a box and whisker plot that shows the wide range of test scores, although most students overall scored well.

**Table 1:** Summary of test scores

	3d (n=18)		Control (n=21)		
	Median	IQR	Median	IQR	p-value
Questions #1 - 8 Ultrasound Principles	7	(5, 7)	7	(6, 7)	0.73
Questions # 9 - 24 Cardiac Anatomy	13	(9, 14)	14	(11, 15)	0.77
Total	19	(14, 21)	20	(16, 22)	0.60

Questions #1 - 8 (Q1-8) to assess ultrasound principles lacked normal distribution in each study arm, but the median for each study arm was 7. The p-value of 0.73 reflected the lack of statistical significance between the two study arms for this section of the multiple-choice test. This was consistent with the initial hypothesis. Questions #9 - 24 (Q9-24) to assess cardiac anatomy again lacked normal distribution but had a median of 13 for the 3d arm and a median of 14 for the control arm. The p-value of 0.77 reflected the lack of statistical significance between the two study arms. This was not consistent with the initial hypothesis that 3d modeling would result in higher test scores than the group that was exposed to conventional methods of explanation.

The mean test scores were higher than theorized. Therefore, a post-hoc power calculation with this new data was assessed, and determined that the study achieved a power of 7.3%. It was not feasible to continue the study, as the class size for the medical students would not provide a large enough sample population.

## Discussion

Ultrasound is a valuable tool that is being integrated into medical education with variable aims and results [6]. Simultaneously learning anatomy and ultrasound imaging is inherently challenging. Utilizing a 3d cardiac model during the ultrasound tutorial to provide improved cardiac anatomic education was not statistically supported in this study. The initial hypothesis that there would be no statistical significance in Q1-8 did hold true to serve as an appropriate internal control for a novel testing method. However, there was no statistical difference between the two study arms in Q9-24, which was not the expected finding. Ultimately, although 3d models were used in the experimental group, the participants were watching video tutorials, which in effect makes this still a 2-dimensional (2d) tutorial. The original plan to have students observe an ultrasound demonstration with standardized patients was not able to be conducted due to health and safety protocols in the setting of coronavirus precautions.

There are many other limitations within this study. It is unknown how each of the study participants scored on their baseline medical school anatomy courses or the number of first and second-year students within each study arm. Additionally, the second-year medical students had been exposed to a one-day ultrasound workshop at the end of their second year of medical school. As this was a required academic exposure, it was not an experience that limited study participation. It did, however, provide the second-year medical students with a possible advantage over the first-year medical students. The two different cardiac ultrasound videos themselves followed the same written script with the intent to prevent significant deviation. The control video taught in a conventional manner had a run time of 8 minutes 41 seconds while the video that included 3d cardiac models had a run time of 11 minutes 31 seconds. This was initially identified as a confounding variable, as the 3d model study arm essentially has more repetitions through the cardiac anatomy images. The test results did not show statistical significance between the two groups so the true effect of this variable remains unknown. The study overall was significantly underpowered as the test scores were higher than predicted for all participants.

Despite the limitations discussed above, this research study offered many positive elements. The testing technique utilized in this study may translate well to future studies. By purposefully not including any orienting text, diagrams, or labels, the study subjects were forced to truly test their skill acquisition from the brief video tutorials. Given that this study was underpowered, it is possible that any statistical effect was not appreciated due to the small size. Further study would be useful.

Albeit the many flaws discussed, there is a growing interest in the integration of bedside ultrasound, anatomy, and models or simulation trainers. There is an abundance of commercially available ultrasound simulation models as well as homemade cost-effective models [7-9]. The numerous innovative approaches to education have demonstrated a theme that students prefer these ultrasound demonstrations. Further research into how to objectively develop that educational approach is needed.

As far as the authors are aware, this is the first study to utilize 3d cardiac models within ultrasound education and the testing methods may provide future utility for similar studies. With the explosion of ultrasound into the medical school curriculum and many specialty fields of training, assessing the most effective way to convey anatomic and physiologic concepts would intuitively benefit from the continued pairing of 3d models with ultrasound imaging.

## Conclusions

Medical school education is increasingly augmented with the inclusion of ultrasound. Although this study did not statistically demonstrate the expected findings, there were several limitations. Further investigation regarding the use of 3d modeling in medical education may have future improved educational benefits.
